# Melatonin’s effect on hair follicles in a goat (*Capra hircus*) animal model

**DOI:** 10.3389/fendo.2024.1361100

**Published:** 2024-04-02

**Authors:** Youjun Rong, Rong Ma, Yanjun Zhang, Zhenhua Guo

**Affiliations:** ^1^ College of Animal Science, Inner Mongolia Agricultural University, Hohhot, China; ^2^ Northern Agriculture and Livestock Husbandry Technical Innovation Center, Chinese Academy of Agricultural Sciences, Hohhot, China; ^3^ Institute of Animal Husbandry, Heilongjiang Academy of Agricultural Sciences, Harbin, China

**Keywords:** alopecia, cashmere, molecular docking, network meta-analysis, single nucleotide polymorphism

## Abstract

**Introduction:**

Melatonin can treat androgenetic alopecia in males. Goats can be used as animal models to study melatonin treatment for human alopecia. In this study, a meta-analysis of melatonin’s effects on goat hair follicles was pursued.

**Methods:**

Literature from the last 20 years was searched in Scopus, Science Direct, Web of Science and PubMed. Melatonin’s effect on goat hair follicles and litter size were performed through a traditional meta-analysis and trial sequential analysis. A network meta-analysis used data from oocyte development to blastocyst. The hair follicle genes regulated by melatonin performed KEGG and PPI. We hypothesized that there are differences in melatonin receptors between different goats, and therefore completed melatonin receptor 1A homology modelling and molecular docking.

**Results:**

The results showed that melatonin did not affect goat primary follicle or litter size. However, there was a positive correlation with secondary follicle growth. The goat *melatonin receptor 1A* SNPs influence melatonin’s functioning. The wild type gene defect *MR1* is a very valuable animal model.

**Discussion:**

Future studies should focus on the relationship between goat SNPs and the effect of embedded melatonin. This study will provide theoretical guidance for the cashmere industry and will be informative for human alopecia research.

## Graphical Abstract

To clarify the suitability of goats as an animal model for melatonin treatment studies in human alopecia, literature from the past 20 years was searched for in Scopus, Science Direct, Web of Science, and PubMed for melatonin’s effects on goat hair follicles, oocyte development *in vitro*, and litter size. Three types of meta-analyses were performed, including traditional meta-analysis, network meta-analysis, and trial sequential analysis. The hair follicle genes regulated by melatonin were analysed by KEGG and PPI. The results showed that melatonin did not affect goat primary follicles and litter size. However, there was a positive correlation with secondary follicle growth. Goat *melatonin receptor 1A* SNPs affect melatonin. The wild-type gene defect of *MR1* is a very valuable animal model. Future studies should focus on the relationship between goat SNPs and the effect of embedded melatonin.
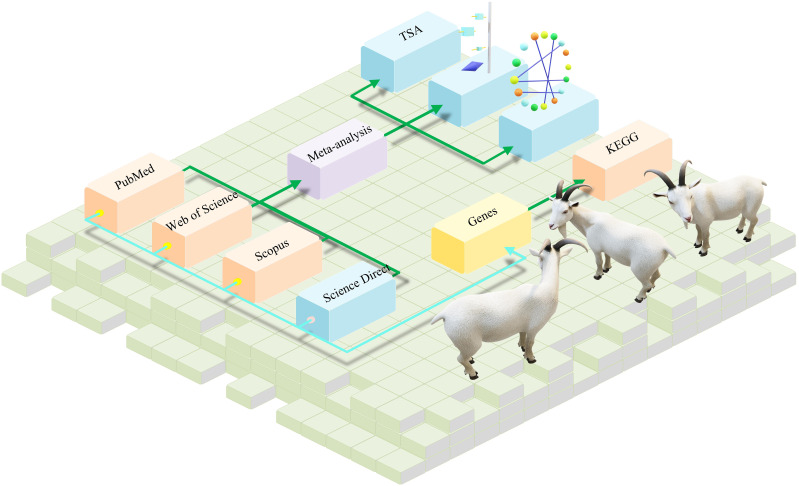

## Highlights

- Melatonin did not affect goat primary follicle and litter size. However, there was a positive correlation with secondary follicle growth.
- Goat melatonin receptor 1A SNPs affects melatonin to exert its function.
- Wild-type gene defect of MR1 is a very valuable animal model.

## Introduction

Alopecia (hair loss) due to endocrine disorders has always plagued humans (1, 2). Animal models for studying hair follicles in humans are usually rats (3), mice (4), guinea pigs (5), rabbits (6), and dogs (7). In addition, the goat *PLP2* (*proteolipid protein 2*) gene expressed only in the inner root sheath, suggesting that it may be associated with alopecia (8). Thus, goat hair follicle research can be applicable for human alopecia (9). It can be used as an animal model for human alopecia research (10).

Melatonin regulates physiological activity in the whole body. Melatonin-associated pathways possibly alleviate radiotherapy-induced alopecia (11). A recent meta-analysis showed evidence to support the use of melatonin to promote scalp hair growth, with melatonin being more effective in men with androgenetic alopecia (12). Moreover, melatonin regulates gene expression in the hair follicles of goats and affects hair follicle growth (13).

Melatonin affects oocyte development in humans (14); there has been a study on goat oocyte development (15). In addition, studies on melatonin, luzindole, and cysteamine mixed effects have also been reported (16, 17), and such research data are suitable for network meta-analysis.

Embedding melatonin under the skin of goats can increase cashmere production (18, 19). Melatonin can increase goat secondary follicle density and does not affect the growth of primary follicle density (18, 20). On the contrary, melatonin significantly reduces primary follicle density in goats (21, 22). It is necessary to analyze the effect of melatonin on goat hair follicles. This study provides theoretical guidance for the cashmere industry.

## Methods

### Database search strategy and study inclusion

**Figure 1:**
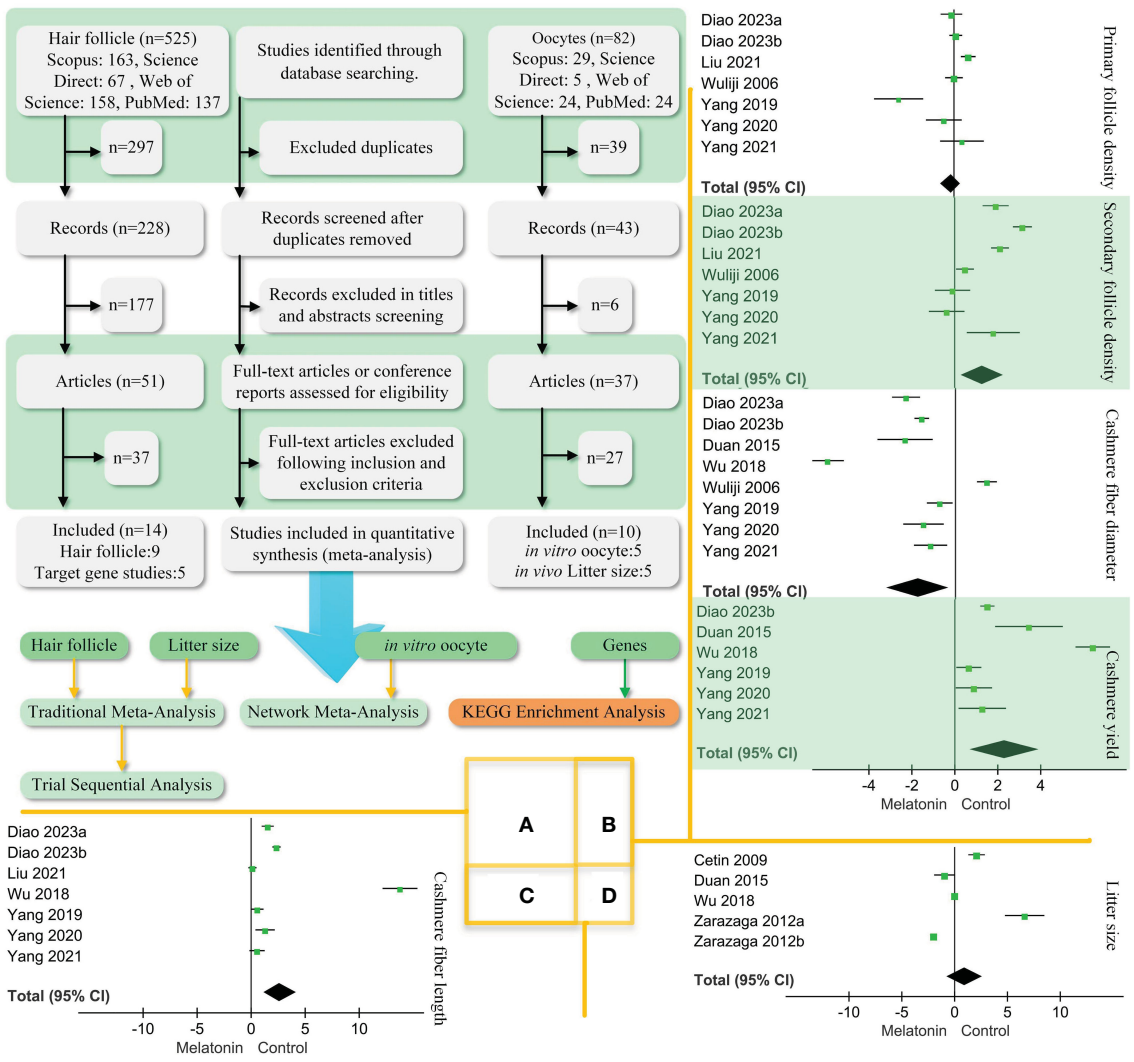
PRISMA diagram of the process of study selection and traditional meta-analysis. **(A)**. We followed the steps to screen the retrieved literature and ended up with 22 articles. Two articles had both hair follicle findings and litter size. Hair follicle, litter size, *in vitro* oocyte, and gene data performance traditional and network meta-analysis. **(B)**. Traditional meta-analysis of follicle density, cashmere fibre diameter, and yield. **(C)**. Traditional meta-analysis of melatonin’s effect on cashmere fibre length. **(D)**. Traditional meta-analysis of melatonin’s effect on goat litter size. Black diamond block represents 95% CI.

Three scholars searched for related studies in Scopus, Science, Web of Science, and PubMed published between 01/01/2003 and 01/09/2023. Two searches were performed. In the first, we searched for effects of melatonin on goat hair follicle growth. The search terms were melatonin AND (hair follicle) AND (goat OR ram OR ewe OR ovine). This search strategy includes hair follicle data as well as data on regulatory genes. In the second search, we looked for effects of melatonin on goat oocyte development. The search keywords were melatonin AND (ovine OR goat OR ewe OR ram) AND (oocyte OR implant). Each of the three scholars worked independently and negotiated any disagreements. Literature mentioned random trials or what the author believes are individuals randomly selected from a population. Literature was included according to the following points: (1) The studied species included goats but were not restricted to goats. (2) The writing was in English. (3) Hair follicle growth or oocyte development was studied in the paper. The process of study inclusion is shown in **Figure 1A**.

### Data extraction

Study data were extracted as continue-type data that included the number of study individuals, observations, and SD (standard deviation) or SE (standard error). SE was converted to SD for extraction. If the data were in figures, the GetData Graph Digitizer (version 2.26) was used to obtain the data (23). When extracting data of melatonin’s effect on gene expression, only the genes mentioned in the study were obtained and the data uploaded to the database were not used.

### Traditional and network meta-analysis

A traditional meta-analysis was used to analyze hair follicle and litter size data. Specifically, primary and secondary hair follicle densities, cashmere yield, cashmere fiber length, and litter size were treated as continuous data. Using Review Manager (version 5.4), the meta-analysis was performed according to the random model. Java (version 1.8.0) was used to perform the trial sequential analysis (TSA) on the above data.

Network meta-analysis data from the development of oocytes to blastocysts were used. R software (version 4.1.2) and the “coda,” “rjag,” and “gemtc” packages in JAGS (Just Another Gibbs Sampler, version 4.3.0) were used for the analysis. The results of the network meta-analysis were landscaped and processed using 3DMax software (version, 2023).

### KEGG enrichment analysis and PPI network analysis

For the genes regulated by melatonin, DAVID (https://david.ncifcrf.gov) was used to procedure the KEGG enrichment analyses. The reference species was chosen as the goat *Capra hircus*. GraphPad Prism (version 9.0.2) was used to visualize the obtained data.

For the same genes regulated by melatonin procedure PPI (protein–protein interaction networks) construction analysis, STRING (https://cn.string-db.org/) was used to establish an original PPI network data. The data were visualized using Cytoscape (version 3.7.2).

### Homology modelling of melatonin receptor 1A 3D structures and molecular docking

To elucidate the reasons for the discrepancies in the data reported in the various studies, we hypothesized that there are differences in melatonin receptors in different goats. SNPs (single-nucleotide polymorphisms) were previously reported to affect cashmere production in goats (24) through *melatonin receptor 1A (* 25). Five SNPs (190, 424, 577, 589 C>T, and 421 T>C) NCBI data (gene number, AF419334) were downloaded. Each mutation was translated into an amino acid sequence, and a homology model was created based on the Swiss model (https://swissmodel.expasy.org/).

The ligand structure of melatonin was downloaded from PubChem. AutoDock (version 4.2.6) conducts protein and ligand docking and estimates the affinity. The docking results were analyzed using PyMOL (version 2. 6. 0).

## Results

### Study selection and characteristics

A total of 607 studies were retrieved, including 525 studies involving hair follicles and 82 studies on oocytes. **Figure 1A** shows that 22 papers were eventually obtained for this study (**Table 1**). The results of effects on goat hair follicle and litter size are listed in two papers that mentioned both hair follicles and litter size (19, 29). Melatonin regulator goat secondary hair follicle genes are listed in **Table 2**. **Table 3** shows melatonin’s effect on goat oocyte development *in vitro*.

**Table 1:** Characteristics of selected studies’ effects of melatonin on goat hair.

	Study and year	Breed	Body weight or age	Embed concentration	Gender	Duration	Number
*1*	#Diao, 2023a (20)	Cashmere	1 day	2 mg/kg every 2 months	NM	2 years	32
*2*	#Diao, 2023b (18)	Cashmere	2, 3, 4, 5, 6, and 7 years	2 mg/kg every 2 months	NM	60 days	180
*3*	#Liu, 2021 (26)	Cashmere	2 years	2 mg/kg every 2 months	Female	1 year	12
*4*	#Wuliji, 2006 (27)	NM	2 years 10 months	18 mg, 6 weeks	Female	4 months	80
*5*	#Yang, 2021 (28)	Mongolian Cashmere	3 years	2 mg/kg every 2 months	Female	2 years	24
*6*	#Yang, 2020 (21)	YiWei White	Ewe 2, 3, and 4 years	2 mg/kg every 2 months	Female	8 months	24
*7*	#Yang, 2019 (22)	Mongolian Arbace	12 days	2 mg/kg every 2 months	NM	1 year	16
*8*	*#Wu, 2018 (19)	YiWei White	37. 2 kg	2 mg/kg every 2 months	Female	1 year	150
*9*	*#Duan, 2015 (29)	YiWei White	2 years	2 mg/kg every 2 months	Female	1 year	18
*10*	*Cetin, 2009 (30)	NM	1.5–2.5 years	18 mg	Female	3 months	80
*11*	*Zarazaga, 2012a (31)	Mediterranean	50 kg	18 mg	Female	7 months	32
*12*	*Zarazaga, 2012b (32)	Payoya Murciano–Granadina	50 kg–80 kg	18 mg	Female Male	3 years	1560

Studies marked with # denote an effect of melatonin on goat hair follicles. Studies marked with * denote effects of melatonin on goat litter size.

**Table 2:** Characteristics of selected studies’ melatonin regulator goat secondary hair follicle genes.

	Study and year	Breed	Age	Concentration	Duration	Detected genes
*1*	Diao, 2023 (20)	Cashmere	1 day	2 mg/kg every 2 months	2 years	*circMPP5, Ki-67, K-14, Wnt-10a, Fn1, FGF2, FGF21, FGFR3, MAPK3*
*2*	Diao, 2023b (18)	Cashmere	2, 3, 4, 5, 6, and 7 years	2 mg/kg every 2 months	60 days	*NFκB, AP-1, COX1, COX3, SOD3, GPX1, NFF2L2, TIMP2, TIMP3, CCL21, CXCL12*
*3*	Liu, 2021 (26)	Cashmere	2 years	2 mg/kg every 2 months	1 year	*Wnt-10b, β-catenin, SFRP1, CHP2, FGF21, NTRK2, FGF14, NFKB1, DLL3, TCHHL1*
*4*	Liu, 2022 (13)	Mongolia White	1 year	2 mg/kg every 2 months	1 year	*PDGFRA, WNT5A, BMPR1A, BMPR2*
*5*	Lu, 2023 (33)	Mongolia Albasi	1 year old	2 mg/kg every 2 months	1 year	*RORα, TCHHL1, β-catenin, SFRP1*
*6*	Wu, 2012 (34)	Mongolia Cashmere	1 year	NM	1 year	*Hoxc13, β-catenin*
*7*	Zhang, 2021 (35)	NM	Secondary hair follicles *in vitro* culture	0 ng/L–5,000 ng/L	0–84 h	*CTNNB1, TCF4, LEF1, C-JUN, C-MYC, CYCLIND1, CDK6, BMP4, RORA, Noggin*

NM, not mentioned.

**Table 3:** Characteristics of selected studies’ melatonin effects on goat oocyte *in vitro*.

	Study and year	Breed	Oocyte collection	Concentration	Stage	IVM medium	Embryo medium	Duration
*1*	Agarwal, 2018 (15)	NM	Puncture	30 ng/ml	IVM	TCM199 foetal bovine serum	Cook Medical	10 days
*2*	Berlinguer, 2009 (16)	Sarda	Ovariectomy/slice	18 mg	Implant 27 days	TCM199 estrous goat serum	SOF	7 days
*3*	Saeedabadi, 2018 (36)	NM	NM	1 µM, 1 nM, 1 pM	IVM	HTCM199 foetal bovine serum	CR1aa	8 days
*4*	Soto-Heras, 2018 (37)	Spanish breeds	Slice	0.01 nM, 1 nM, 0.1 µM, 1 mM	IVM	TCM199 foetal bovine serum	SOF	8 days
*5*	Soto-Heras, 2019 (17)	NM	Slice	0.1 μM	IVM	TCM199 foetal bovine serum	SOF	8 days

NM, not mentioned.

### Traditional meta-analysis

**Figures 1B, D** show that melatonin did not correlate with primary follicle density and litter size in goats. Embedding melatonin under the skin of goats was positively correlated with secondary follicle density (SMD = 1.27, 95% CI = 0.31–2.22; *p* < 0. 001), cashmere yield (SMD = 2.35, 95% CI = 0.74–3.95; *p* < 0. 001), and fiber length (**Figure 1C**, SMD = 2.7, 95% CI = 1.14–4.25; *p* < 0. 001). However, there was a negative correlation to cashmere fiber diameter (SMD = −1.70, 95% CI = −3.1–(−0.31); *p* < 0. 001).

### Network meta-analysis and TSA

**Figure 2:**
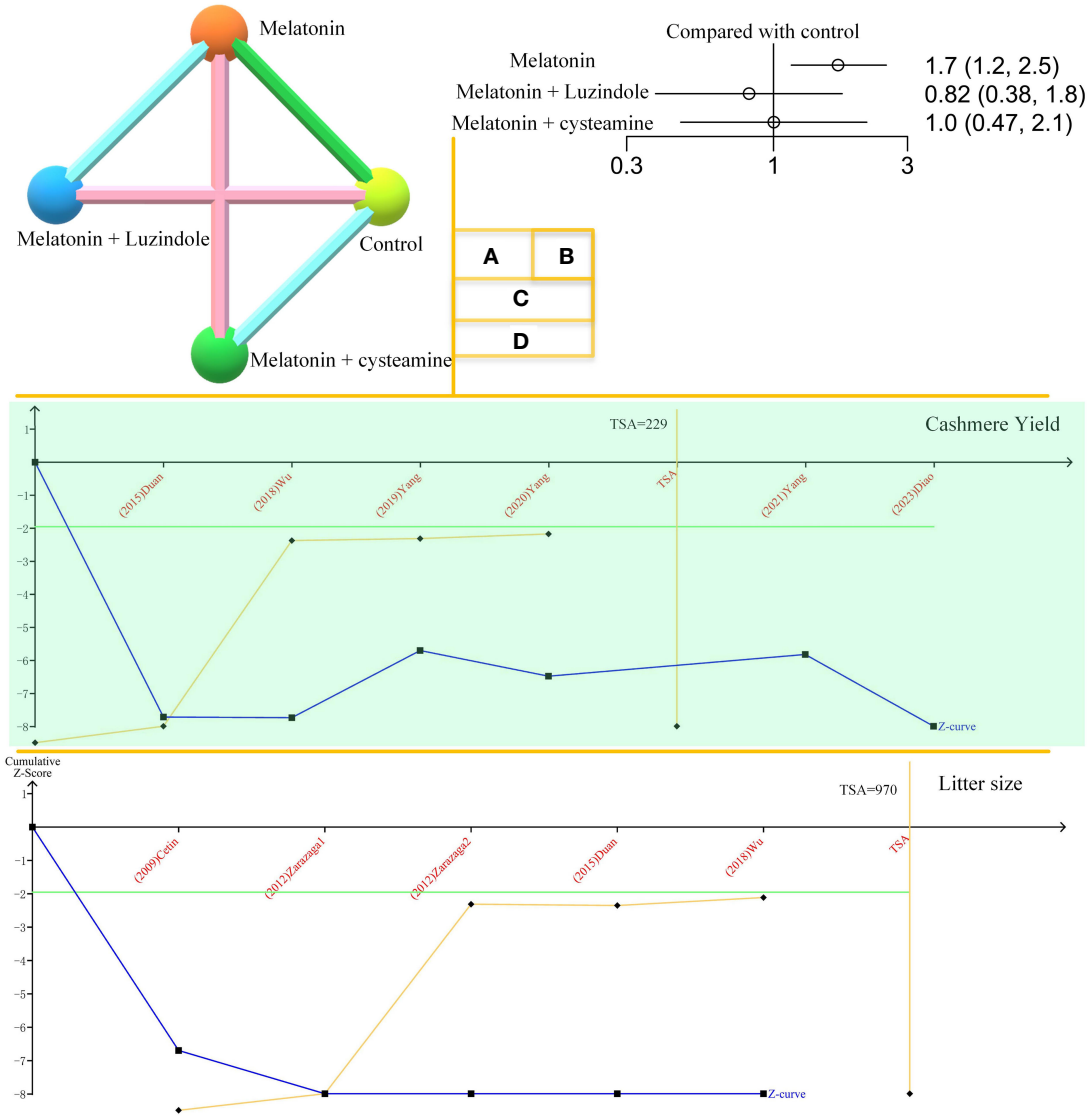
Network meta-analysis and TSA. **(A)**. Network plot of melatonin’s effects on goat oocyte development *in vitro*. **(B)**. Forest plot of melatonin’s effects on goat oocyte development *in vitro*. **(C)**. Trial sequential analysis (TSA) of cashmere yield. The orange line represents the horizontal line in the traditional sense. The TSA mathematical expected value is 229. **(D)**. TSA of melatonin’s effect on goat litter size.

The results of this network meta-analysis are shown in **Figures 2A, B**. This includes three two-arm and two three-arm studies. Adding melatonin to the *in vitro* culture medium was positively correlated with the development of goat oocytes to blastocysts. Neither melatonin plus luzindole nor cysteamine showed correlations in this process.

The TSA results for cashmere yield are shown in **Figure 2C**. The amount of information required for TSA is 229, and the Z-curve crosses the monitoring boundary. Melatonin’s effects on cashmere yield in goats are considered credible. **Figure 2D** shows the effect of melatonin on goat litter size. The curve crosses the monitoring boundary. However, the required information size was not reached. The results of the effect of melatonin on cashmere production in goats are considered credible. However, more research is needed to support this conclusion.

### Gene enrichment analysis and PPI network analysis

**Figure 3:**
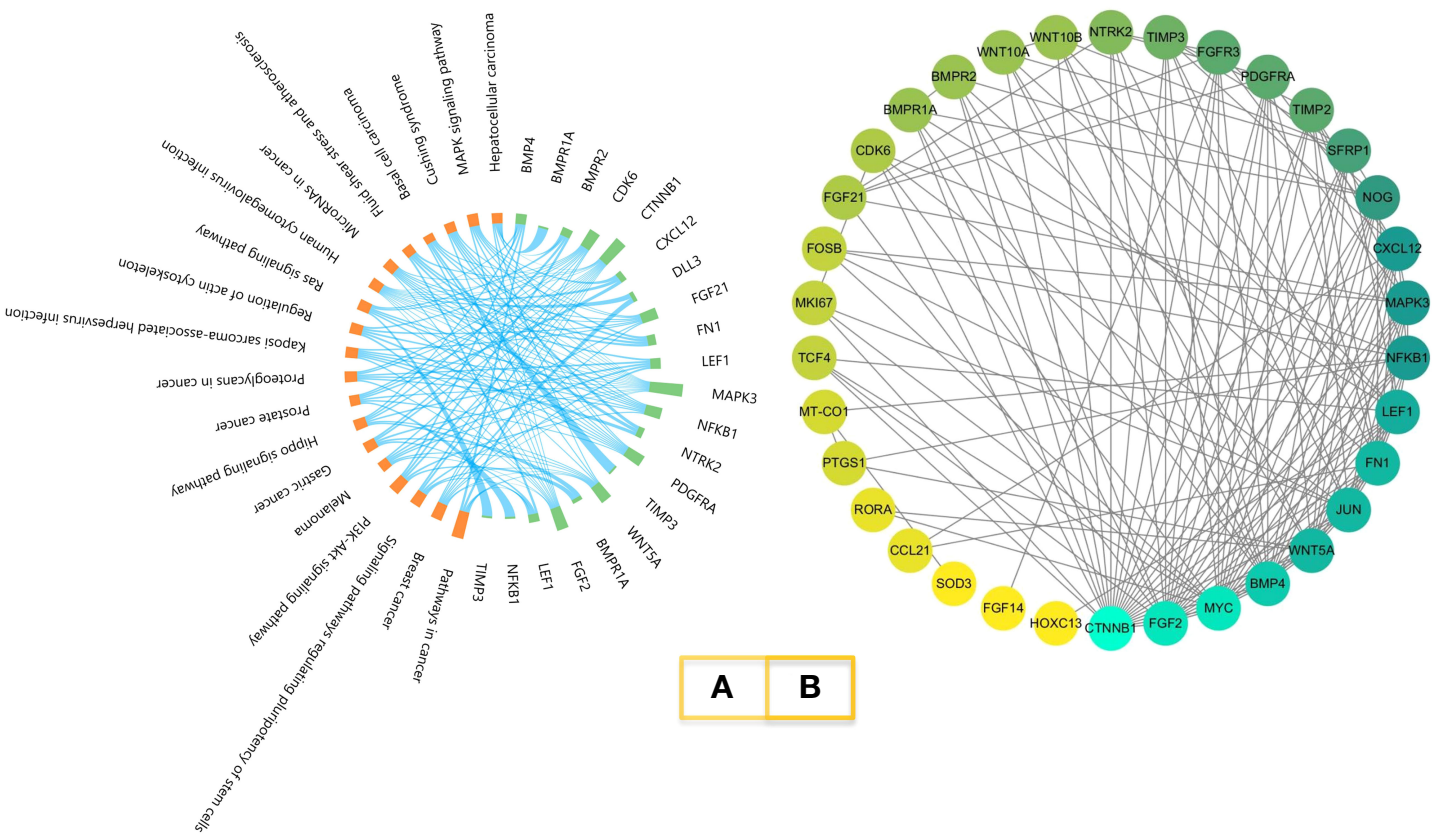
KEGG enrichment and PPI network. **(A)**. Melatonin-regulated gene KEGG enrichment. Orange represents the pathway; green represents the genes. **(B)**. PPI network of melatonin-regulated genes. Proteins are ordered by the numbers of interactions.

The KEGG results of melatonin regulator genes are shown in **Figure 3A**. The 19 pathways with the highest *p*-values were selected, which included the MAPK signaling pathway, *BMP* (*bone morphogenetic proteins*) genes, and receptors, which are the pathways we have been focusing on (38). The results of the PPI network analysis are shown in **Figure 3B**. BMP proteins and receptor proteins are also included.

### Homology modelling of melatonin receptor 1A 3D structures and molecular docking

**Figure 4:**
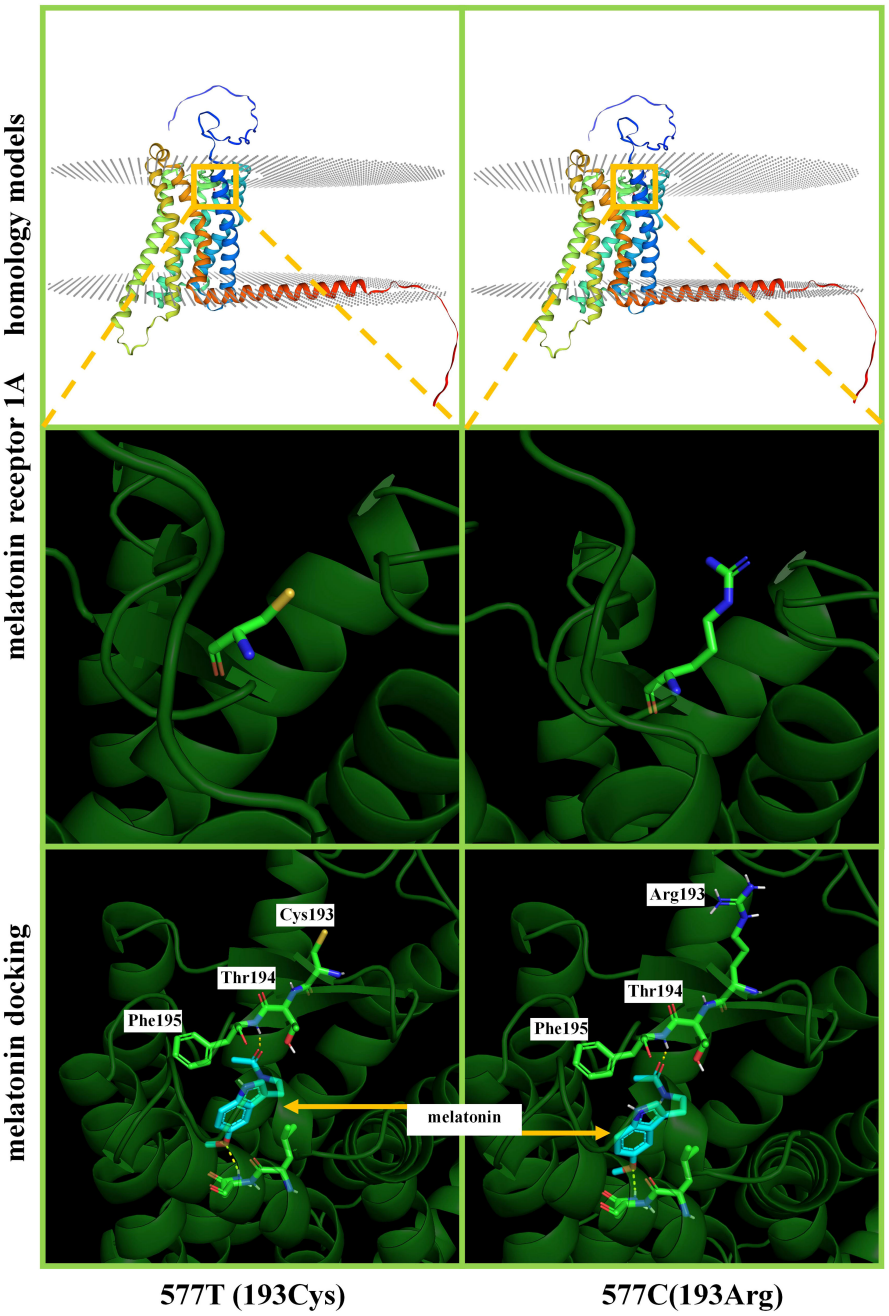
Homology modelling and ligand docking of melatonin receptor 1A structure. 577T and 577C represent the T>C mutation, which results in the amino acid chain 193Cys>Arg. The top and middle show that this mutation does not cause a change in the higher structure of the protein. Below is a magnification of melatonin receptor 1A structures and ligand docking binding pocket; the hydrogen bonding site is 195Phe, which does not involve the 193rd position on the amino acid chain.

In total, five SNPs (190, 424, 577, and 589 C>T and 421 T>C) were analyzed individually. The SNP 589 C>T has a terminator at amino acid 197 and is not able to translate a complete protein. SNPs 190, 424, 577 C>T, and 421 T>C do not affect the higher structure of melatonin receptor 1A. SNP 577 C>T is closer to the melatonin docking position, and thus we were simulating ligand docking. **Figure 4** shows melatonin docking of 577 C (193Cys) and 577 T (193Arg), with no change in the docking pocket. A total of 195 Phe hydrogen bonds were 2.1 and 2.3 A. The binding energies were 6.53 and 6.35, respectively.

## Discussion

Androgenetic alopecia is a widespread problem, and hair follicle growth studies are key to treating alopecia (39, 40). Melatonin may be a potential treatment for androgenetic alopecia (12). Rats (3), mice (4), guinea pigs (5), rabbits (6), and dogs (7) are animal models for studying human hair follicles. Comparative studies on the impact of diacylglycerol O-acyltransferase 1 (DGAT1) on mouse and dog alopecia suggest that mice may be an especially sensitive species (41).

### Melatonin affects hair regeneration

Melatonin promotes hair follicle growth in humans (12) and goats. Primary hair follicles are generally considered the hair that has already developed. The secondary follicles are the newly grown hairs. The results of the present study showed that there was no correlation between melatonin and goat primary follicle density. However, melatonin was positively correlated with goat secondary follicle density. This suggests that melatonin can promote hair regrowth.

Melatonin promotes the expression of *MTNR1A* (*melatonin receptor 1A*) in human and rex rabbit hair follicles (42, 43) and also enhances the expression of goat *Wnt10b* and *beta-catenin (* 26). The signaling pathways involved in the regulation of hair follicle growth by melatonin are the PI3K/AKT signaling (43), Hippo, TGF-beta, MAPK signaling (13), and AKT/GSK3beta/beta-catenin signaling pathways (44). The results of the present study suggest that the MAPK signaling pathway is also at the forefront of KEGG enrichment. We have been focusing on the effects of the signaling pathways on reproduction (38). In addition, oral melatonin exerts a systemic effect on all cells, tissues, and organs, and it plays a key regulatory role in female reproduction (45). Therefore, the effect of embedded melatonin on goat litter size was considered in this study.

### Melatonin and goat litter size

Adding melatonin *in vitro* can promote oocyte development in humans (14), mice (46), bovines (47), sheep (48), and swine (49). Our network meta-analysis results also showed that melatonin could promote goat oocyte development *in vitro*. However, melatonin did not affect goat litter size. There are three possible reasons for this. First, embedded melatonin enters the bloodstream then passes through the blood-follicle barrier (BFB), where the concentration changes dramatically. Second, melatonin affects sheep litter size, not through direct action, but by regulating hormonal changes in the whole body (48). Third, melatonin may play different roles at different stages of oocyte development. An example of a similarity is that follistatin inhibits oocyte maturation before meeting sperm. However, it promotes zygote development to blastocysts after fertilization (38).

We previously reported that melatonin can be positively correlated with sheep litter size (48). However, the results of our analysis did not correlate with goat litter size. We believe that this is because the purposes of the experiments were different and the treatments of embedded melatonin were different. Goats embedded with melatonin produced more cashmere if it was embedded for 6 months at 2 mg/kg every 2 months (18, 28). Sheep were generally embedded with melatonin 35 days to obtain more lambs (48).

### Comparative analysis of melatonin on human and goat hair follicle growth promotion

**Figure 5:**
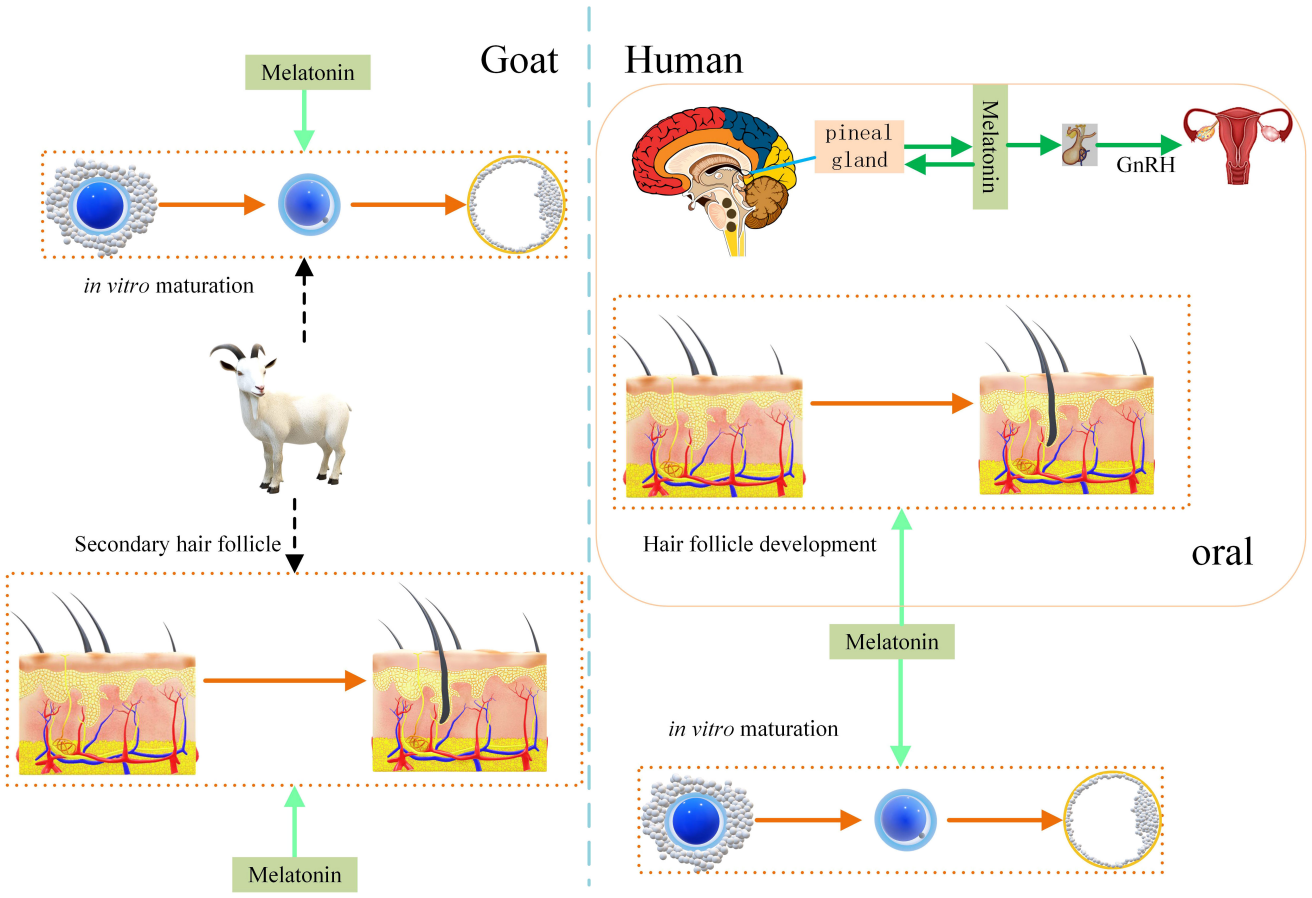
Effect of melatonin on hair follicle growth in humans and goats. Melatonin promotes hair follicle growth in humans and secondary hair follicle growth in goats. Melatonin promotes human and goat oocyte competence under *in vitro* culture conditions. That increase development rate of oocyte to embryo. Melatonin regulates the human reproductive system.

**Figure 5** compares the promotion of hair follicles by melatonin in humans and goats, which can provide more reference for goats as a model animal. Melatonin regulates both reproduction and alopecia in humans (12, 45). Promotion of secondary hair follicle growth has also been obtained in studies on goats (13). Melatonin plays a promotional role during *in vitro* maturation of goat and human oocytes (14, 17). Appropriate oral administration melatonin can enhance whole-body physiology. For example, it improves sleep and boosts immunity. However, excessive melatonin may lead to depression (50).

### Effect of goat *melatonin receptor 1A* SNPs on experimental results

Melatonin regulates the body’s biological clock and sleep rhythms by binding to receptors. Melatonin receptors 1A and 1B (MT1 and MT2) are members of the family of G protein-coupled receptors (GPCRs)) (51). MT3 (quinone reductase 2) has low binding affinity with melatonin (52). *Melatonin receptor 1A* and *1B* genes are differentially expressed at different locations in the brain and may perform different physiological functions (53). We investigated the SNPs and ligand docking of melatonin receptor 1A and found that 589 C>T has a terminator at the 197th amino acid. That leads to the loss of function of melatonin receptor 1A, which may lead to uncertain results if such goat individuals are mixed into experimental groups. This could also explain the inclusion of literature where opposite results were seen. Future studies should screen goat SNPs before embedding melatonin. More studies focus on SNPs of human melatonin 1A receptors (54). Goat wild-type genes defective for *MR1* can be used as an animal model to study alopecia (24).

## Limitations

Melatonin can interact with enzymes, molecular channels, transporters, and signaling molecules to perform physiological functions (45, 55). This study only considered *melatonin receptor 1A*, and this has some limitations. In addition, there is very limited research on human secondary follicles and melatonin. This will also be a limitation for goats as a research model for human alopecia.

Intestinal melatonin concentrations were 400 times higher than those of the pineal gland (56), with uncertainty in the results when the effect of intestinal flora was ignored. For our inclusion study, both experimental and control groups were under the same feeding management conditions, and we defaulted to a negligible effect of intestinal flora. For the same reason, whether oral administration of melatonin in humans and embedded melatonin behind the ears of goats would have the same effect also needs further investigation because oral magnesium sulphate administration (57) can achieve completely different pharmacological effects than injection (58).

## Conclusion

Goats can be used as an animal model for human alopecia research. Melatonin does not affect goat primary follicle or litter size. However, there is a positive correlation with secondary follicle growth. Melatonin was positively correlated with the development competence of goat oocytes. Goat *melatonin receptor 1A* SNPs affect melatonin to exert its function. The wild-type gene defect of MR1 is a valuable animal model. Future studies should focus on the relationship between goat SNPs and the effect of embedded melatonin. This study can provide a reference for improving cashmere production and a suggestion for animal models of human alopecia.

## Data availability statement

The original contributions presented in the study are included in the article/supplementary materials. Further inquiries can be directed to the corresponding authors.

## Author contributions

YR: Data curation, Writing – original draft. RM: Data curation, Writing – original draft. YZ: Funding acquisition, Writing – original draft. ZG: Writing – original draft, Writing – review & editing.
